# Stakeholders’ perceptions of transferability criteria for health promotion interventions: a case study

**DOI:** 10.1186/1471-2458-14-1134

**Published:** 2014-11-04

**Authors:** Justine Trompette, Joëlle Kivits, Laetitia Minary, Linda Cambon, François Alla

**Affiliations:** EA 4360 Apemac, Faculté de médecine, Université de Lorraine, 54250 Vandœuvre-lès-Nancy, France; Inserm, CIC-EC, Centre hospitalier universitaire, 54000 Nancy, France; Faculté de Médecine, Ecole de Santé Publique, 9 avenue de la Forêt de Haye – BP 184, Université de Lorraine, 54505 Vandœuvre-lès-Nancy, France

**Keywords:** Transferability, Health promotion, Intervention, Implementation, Evidence-based health promotion

## Abstract

**Background:**

The effects of health promotion interventions are the result not only of the interventions themselves, but also of the contexts in which they unfold. The objective of this study was to analyze, through stakeholders’ discourse, the characteristics of an intervention that can influence its outcomes.

**Methods:**

This case study was based on semi-structured interviews with health promotion stakeholders involved in a regional program (PRALIMAP). General hypotheses on transferability and on how the intervention is presumed to produce its effects were used to construct an interview guide. Interviews were analyzed using thematic coding.

**Results:**

Twenty-three stakeholders were interviewed. Results showed stakeholders made few references to population and environment characteristics. Three themes emerged as significant for the stakeholders: implementation modalities and methodology, modalities used to mobilize actors; and transferability-promoting factors and barriers.

**Conclusion:**

Our work contributes to a better understanding not only of transferability factors, but also of stakeholders’ perceptions of them, which are just as important, because those perceptions themselves are a factor in mobilization of actors, implementation, and transferability.

## Background

Health promotion interventions combine actions on public policy, on physical and social environments, and on behaviours. They give people the means to have more control over their own health and to improve it [1–3]. Such interventions are complex [4–8] because they are made up of a number of components (population, environment, and intervention factors, individuals’ representations of their health, acceptance of interventions by all those concerned, available resources, etc.) that may act both independently and interdependently. Complexity also arises in the interaction between the interventions and the contexts within which they unfold [9].

These factors affect the transferability of health promotion interventions. An intervention’s transferability is defined as the extent to which its effects in a given setting can be observed in another setting [10]. It differs from applicability, defined as the extent to which an intervention’s process can be implemented in another setting [10]. Transferability depends on implementation conditions and on the interaction between the intervention and the context into which it is inserted [1, 11]. It is a key consideration in ensuring *a priori* that an intervention’s outcomes will be the same when it is transferred from one context to another, or from research to real life. It is therefore a major issue in the area of health promotion. Indeed, even if an intervention has demonstrated its effectiveness in a given setting, the observed effects are rarely identical in another setting; the intervention itself may be applicable, but it may generate other effects than those seen in the primary intervention [11]. Many transferability factors were identified in a recent review [11], related to population, implementation process, stakeholders, environment, or specific health problems. These factors were also generally identified as external validity or applicability factors and included in implementation models and tools in the health promotion and healthcare fields [2, 12–21]. A practical tool using these criteria has been proposed to assess the transferability of health promotion interventions [22].

While we can identify transferability factors and formulate criteria for successful transferability, we have yet to understand more fully what intervention characteristics influence outcomes, how they produce their effects, and whether they are context-specific or intervention-specific. We can hypothesize that knowledge about these characteristics and how they influence the intervention outcomes will enhance implementation conditions and criteria for successful transferability.

Exploring transferability and associated criteria involves scrutinizing what actually happens when an intervention is implemented in the field. In this respect, stakeholders’ perspectives are an important and rich source of knowledge [23–25]. For one thing, the significance actors give to their roles and actions within the intervention sheds light on intervention processes and mechanisms. What aspects of the implementation do actors perceive as important for ensuring the success of the intervention? Do actors consider and integrate transferability factors when implementing the intervention? For another, stakeholders’ perceptions provide a dynamic view of the intervention that is crucial in understanding transferability components and determinants. Indeed, the actors’ perceptions of the intervention influence their decision to transfer it or not, and, if it is transferred, how they adapt it to the new context.

The objective of this study was to analyze, through stakeholders’ discourse, the characteristics of interventions that can influence intervention outcomes, in order to provide contributive data on the implementation process and on intervention transferability.

## Methods

We selected one health promotion intervention as a case study for exploring stakeholders’ experiences of intervention implementation and perceptions of transferability criteria. The case study methodology was preferred, as it enabled us to make more general assumptions regarding the topic being studied, transferability, based on the description and analysis of one case—in this study, a health promotion program [26, 27].

The selected case was PRALIMAP [28], an overweight and obesity prevention program for adolescents, in which the same intervention was implemented in 24 public high schools in the Lorraine region (north-eastern France). It involved a large number of stakeholders: 1) project sponsors, who planned the intervention, monitored its implementation, and ensured its funding; 2) intermediary participants, who coordinated the intervention in high schools and ensured successful implementation; and 3) field participants, who delivered the intervention to the target population in the high schools concerned.

The case study was based on semi-structured interviews with PRALIMAP stakeholders.

### Data collection tools

We constructed an interview guide based on the ASTAIRE tool, which lists 23 transferability criteria for health promotion interventions [22]. First, we used these criteria to formulate hypotheses about how intervention components, such as the environment, might affect the intervention results. Twenty hypotheses were generated in this way: 12 dealing with population characteristics, seven with intervention characteristics, and one with the environment (Table 1). From these hypotheses, we drafted an interview guide with questions on factors related to population characteristics and factors related to intervention characteristics. In framing the questions, we intentionally avoided any explicit mention of the ASTAIRE tool or transferability criteria, and we formulated them to be understandable by persons untrained in the field of health promotion. This enabled us to capture the elements that, according to stakeholders, influenced the results of the intervention.

**Table 1:** **Hypotheses and ASTAIRE criteria regarding how environment, intervention and population characteristics might affect outcomes**

Research hypotheses	ASTAIRE tool criteria*
**Hypotheses related to the population**	**ASTAIRE criteria related to the population**
l. People’s perceptions of their own health needs will influence their participation in the action and/or adherence to the behaviour.	1. The epidemiologic and sociodemographic characteristics of the recipient population
2. People’s representations of health (perception, norms, vulnerability, importance) will influence their participation in the action and/or adherence to the behaviour.	2. The cognitive, cultural, social, and educational characteristics of the recipient population
3. People’s perceptions of an intervention’s acceptability will influence their participation in the action and/or adherence to the behaviour.	2. The cognitive, cultural, social, and educational characteristics of the recipient population
4. People’s perceptions regarding the control they have over their own behaviour will influence their participation in the action and/or adherence to the behaviour.	2. The cognitive, cultural, social, and educational characteristics of the recipient population
5. People’s relationships with norms (social and health) will influence their participation in the action and/or adherence to the behaviour.	2. The cognitive, cultural, social, and educational characteristics of the recipient population
6. Each person’s own experience and history/the collective experience and history of a group will influence his/her/its participation in the action and/or adherence to the behaviour.	2. The cognitive, cultural, social, and educational characteristics of the recipient population
7. People’s interest in an intervention will influence their motivation to participate in the action/their adherence to the behaviour.	3. The type of motivation in the intervention’s recipient population
	6. The recipients’ perceptions of the intervention’s utility
	7. The demand coming from the population
	8. The population’s perceptions of their own health needs
	10. The degree of involvement of recipients
8. The climate of trust between an intervention’s providers and beneficiaries will influence people’s participation in the action.	5. The climate of trust between providers and recipients
9. The population’s participation in the action will influence individuals’ adherence to the behaviour.	9. The level of participation among participants
10. The population’s participation in the action will influence the result of the action and/or the intervention.	9. The level of participation among participants
	10. The degree of involvement of recipients
11. The population’s adherence to the behaviour – to the message being promoted – will influence the result of the action and/or the intervention.	*Supplementary hypothesis*
12. Theories 1 to 7 will influence the operationalization of the intervention.	*Supplementary hypothesis*
**Hypotheses related to the characteristics of the intervention**	**ASTAIRE criteria related to the characteristics of the intervention**
13. The skills of those implementing the intervention/the participants’ perceptions of the intervention’s utility/the intervention’s acceptability to the participants/the modalities used to mobilize actors will influence the partnerships (type, number, etc.) and/or the continuous adaptation of the intervention.	17. The skills/capacities of the providers and of the project leader
	18. The providers’ belief in the utility of the intervention
	19. The acceptability to the intervention’s providers
	20. The providers’ mobilization
14. Providing support to those involved in transferring the action will enable (or foster) its continuous adaptation/will influence the intervention’s implementation.	22. Adaptations can be (or were able to be) made to the primary intervention in the replica context without altering its fundamental nature.
	23. The primary intervention has prepared and provided all the elements needed for its transfer. A knowledge transfer process exists in the replica setting.
15. The intervention modalities (or methodology) used (strategies, action plan, communication) will influence the intervention’s implementation.	21. The intervention methods
16. The resources (material, financial, and human) available for the intervention will influence the intervention’s implementation/the accessibility (geographic, financial, and sociocultural) of the action to people.	4. The accessibility of the intervention
	16. The resources for the intervention
17. Antecedents or prior interventions, synergistic or antagonistic, will influence the intervention’s implementation/foster partnerships.	13. Other elements of the implementation context
	14. The partners enlisted for the intervention
18. Partnerships will foster the intervention’s implementation.	14. The partners enlisted for the intervention
19. The intervention’s implementation will influence its results.	*Supplementary hypothesis*
**Hypotheses related to the environment**	**ASTAIRE criteria related to the environment**
20. The intervention’s institutional environment (political will, institutional support, etc.) will influence the intervention’s implementation/the resources available/the potential partnerships.	12. The institutional environment directly influencing the intervention
	13. Other elements of the implementation context
	14. The partners enlisted for the intervention

*The numbers refer to the criterion number in the ASTAIRE tool [22].

### Data collection and analysis

The data were collected between February and April 2013.

We interviewed all three categories (sponsors, intermediary participants and field participants) of stakeholders of the PRALIMAP intervention who had participated in either the first wave in 2006 or the second in 2008, or both. All 24 high schools were contacted; in 11 of them, some of the stakeholders who had participated in PRALIMAP were still working. Of these 11 schools, seven agreed to participate in this research. Stakeholders in the schools were invited for an interview.

The interviews, lasting 15 to 75 minutes, were all recorded, with the exception of one for which the interviewee did not give consent. The recordings were transcribed verbatim and content analysis techniques were applied to the transcriptions [29].

Analysis was conducted in two steps. The first consisted in coding the interviews according to the 20 hypotheses. We developed an analytical grid to identify and count the references made by participants to different intervention and population characteristics that influenced outcomes. In the second, we used classical thematic coding to locate in the interview transcripts meaningful elements of transferability that were not present in the hypotheses, but that emerged from stakeholders’ experience of the PRALIMAP program.

Two researchers (JT, JK) conducted the data analysis. The data were coded and analyzed using NVivo 10 qualitative analysis software.

### Ethical issues

The *Comité de protection des personnes* (French institutional review board) certified that this observational study did not fall under the law on the protection of human subjects, as it did not involve individual subjects or identifying health data. Thus, according to French regulations, the study did not require institutional review board approval. All professionals who participated provided consent.

## Results

We conducted 23 semi-structured interviews (five sponsors, two intermediary participants, 16 field participants). The characteristics of interviewed stakeholders are shown in Table 2.

**Table 2:** **Characteristics of stakeholders interviewed between February and April 2013**

	School reference	Gender	Position	Profession
S1*	1 to 7	Woman	Sponsor	Project manager
S2	1 to 7	Woman	Sponsor	Project manager
S3	1 to 7	Woman	Sponsor	General manager
S4	1 to 7	Woman	Sponsor	Nurse and technical consultant
S5	1 to 7	Man	Sponsor	General manager
I1**	1 to 7	Woman	Intermediary participant	Project manager
I2	1 to 7	Woman	Intermediary participant	Project manager
F1***	1	Man	Field participant	Food manager
F2	1	Man	Field participant	Educational counselor
F3	5	Woman	Field participant	Sports teacher
F4	5	Woman	Field participant	School nurse
F5	4	Woman	Field participant	Educational counselor
F6	4	Woman	Field participant	School nurse
F7	4	Woman	Field participant	Teacher
F8	1	Woman	Field participant	Teacher
F9	4	Man	Field participant	Principal
F10	7	Woman	Field participant	School nurse
F11	6	Woman	Field participant	School nurse
F12	2	Woman	Field participant	School nurse
F13	2	Woman	Field participant	Food manager
F14	2	Woman	Field participant	Educational counselor
F15	3	Woman	Field participant	School nurse
F16	5	Man	Field participant	Cook

*S1: Sponsor 1.

**I1: Intermediary participant.

***F1: Field participant.

Textual data were analyzed using a generic thematic analysis method. We counted the number of references made by interviewees to how environment, implementation, and population characteristics had influenced intervention outcomes (Table 3).

**Table 3:** **Number of references to transferability hypotheses by the various categories of PRALIMAP participants**

	Number of references* to hypotheses by the interviewees		
	Sponsor (N = 5)	Intermediary participant (N = 2)	Field participant (N = 16)
**Hypotheses**			
*Population characteristics*			
1. People’s perceptions of their own health needs	0	0	0
2. People’s representations of health	0	0	0
3. Acceptability of the intervention	5	1	10
4. Perception regarding the control over their behaviour	0	0	0
5. Relationship with norms	0	0	1
6. Interest in an intervention/motivation	0	1	2
Interest in an intervention/adherence behaviour	0	0	0
7. Experience and history	0	0	0
Collective experience and history of a group	0	0	0
8. Climate of trust (intervention’s providers/beneficiaries)	1	1	1
9. Population’s participation in the action/individuals’ adherence	0	0	0
10. Population’s participation in the intervention/result	0	0	0
11. Population’s adherence/result	0	0	0
12. 1–7 influences the implementation	0	0	0
NH. Playful dimension	6	1	7
*Implementation characteristics*			
13. Stakeholder’s skills	37	26	65
Stakeholder’s perception	23	15	48
Acceptance of the intervention by stakeholders	15	10	18
Procedures for mobilizing stakeholders	54	28	104
14. Support for transfer adaptation	0	0	0
Support during transfer implementation	15	11	54
15. Intervention modalities	109	55	205
16. Implementation resources	11	2	25
Resource accessibility	4	0	2
17. Background and implementation	5	1	3
Background and partnerships	7	6	3
18. Partnerships and implementation	23	15	19
19. Intervention’s implementation/results	0	0	0
NH. Team stability	6	9	11
*Environmental characteristics*			
20. Institutional environment and implementation	15	2	5
Institutional environment and resources	5	1	0
Institutional environment and partnerships	2	0	0

*These references were collected during the semi-structured interviews conducted between February and April 2013.

NH: New hypothesis.

Although interviews included questions on population and environment characteristics, stakeholders made few references to these characteristics and did not perceive them as major influences on the intervention and its outcomes. By contrast, references to the implementation of the intervention and its characteristics elicited a variety of reactions and were more fully developed by interview participants. Hence, implementation characteristics were submitted to further thematic analyses, which are presented below.

Three themes emerged as significant for the stakeholders: implementation modalities and methodology; modalities used to mobilize actors; and transferability-promoting factors and barriers.

### Implementation modalities: communication

Among the implementation modalities mentioned by the participants, communication was particularly important. From the stakeholders’ standpoint, it was a key element of project success. They mentioned three aspects of communication: the importance of multidisciplinary work, stakeholders’ communication skills, and overall project communication.

#### Multidisciplinary work

Generally, participants reported working on multidisciplinary teams. However, the terms used in relation to this work modality varied depending on the stakeholder’s specific profile. Thus, sponsors and intermediary participants employed terms such as “coordinating committee” and “steering committee”, while field participants talked about “teamwork” in the form of meetings. Multidisciplinary teamwork reflected the communication dynamics among stakeholders involved in implementing the intervention.

*S3: ’… We had coordination committees, steering committees; the roles were defined and very clear. We were responsible for all coordination, plus financial management, communication; all the finances passed through us, all the invoices. …We also had several cross-disciplinary groups working to develop benchmarks….’*

*I2: ‘Within the institution, we had two or three [meetings] during the year, plus the PRALIMAP meetings in Nancy, which, as far as I can remember, also took place twice a year. The meetings were all different, as there were meetings for the nurses with doctors, etc., as well as meetings for teachers, managers, administrators, etc.’*

The intermediary participants and the intervention sponsors indicated that the routine-based operational mode was already part of their practices. However, the process was new for the field participants, who were accustomed to working in their field of expertise (school clinic, canteen, education, etc.) without needing to interact with other areas. The multidisciplinary working processes thus became particularly popular among the field participant group, who continued to work this way afterward.

*JT: ‘Do you think these meetings were important for the implementation?*

*F13: Yes, yes, yes. They are crucial, because this is where we get to know just a little bit of what everyone does. Otherwise it’s up to each individual… that’s the way it is normally. We could say that everyone used to work individually. No, no. It was good, it was essential. Plus, everyone gets the correct information.’*

#### Stakeholders’ skills

Communication was discussed not only as a key component of implementation, but also as a skill for participants, especially the ability to work in a team. The strong links between these aspects were noted—in particular, the fact that each participant’s ability to work as part of a team contributed to the quality of communication and of the multidisciplinary work involved.

Indeed, the ability to work in teams was considered important because it influenced the dynamics of communication around the intervention. If relations between members of the same team were so poor that they could not work together, it could have had a direct impact on the implementation and therefore on the result of the intervention.

*I1: ‘So if there really is a result to take into account, I think it’s on the team level, when it comes to its internal functioning, to the method used to work together. Some people, who had been solely responsible for “nutrition training and environment,” found themselves having to work together with the others. The manager, who is usually alone in his office handling things, etc., here had to interact with the rest of the team in order to be able to put things in place, namely to organize the PRALIMAP program. So I think that taking into account the way we work together is important. This came out at many institutions. I remember this very well because I often heard, “Oh, you’ve just nailed it! It’s not always pleasant to hear, but at the same time you’ve allowed us to move forward, so…’.*

*S3: ‘These types of projects can also be occasions for internal score-settling, and this is one way for someone to express dissatisfaction, but it has nothing to do with your project.’*

Multidisciplinary meetings, more or less regular depending on the school, provided a means to readjust protocols within each institution to best meet the objectives of PRALIMAP.

#### Overall communication for the project

This type of communication, managed by the sponsors, targeted specific field participants. Three examples were highlighted: the intervention implementation meeting, interim meetings, and a meeting for the final presentation on outcomes.

Some field participants stressed that the implementation meeting, the project presentation, and their own commitment and that of their colleagues were all key factors in the intervention. They said it was important for them to have a clear vision of the sponsors’ goals and expectations presented in accessible language. Field participants therefore found themselves participating in sponsors’ and intermediary participants’ routines, a new operational mode for them.

Interim meetings, during which reports were presented on the intervention’s outcomes and consequences, were held at the end of each school year. Some field participants saw these meetings as essential to sum things up, as well as to readjust and strengthen their motivation to participate. Others saw the meetings as not accurate enough and lacking in concrete data concerning their own institutions.

A final meeting was held to present the results at the end of the intervention. Both the field and intermediary participants expressed the importance of getting and giving feedback on the action’s outcomes.

### Impact of top-down approaches to intervention

Regarding the means used to involve participants, interviewees discussed whether the intervention was imposed or not, and the motivation, interest, and degree of involvement of the school’s management. The approach used had an influence on the intervention within schools and on stakeholder involvement, especially in the case of field participants.

#### Ownership

PRALIMAP was part of a French national policy launched in 2001, the National Health and Nutrition Program (NHNP) [30]. This was a public health program aimed at improving population health status by acting on one of its major determinants, nutrition [31]. The sponsor participants said they had implemented this intervention at the request of the regional political authority. The sponsors therefore had a professional interest in setting up this project.

Meanwhile, in follow-up to a pre-existing partnership, the intermediary participants had been approached regarding the development of an educational tool to promote healthy eating, so they had more solid training in the nutrition area. These interviewees reported having both a personal interest (in the theme of nutrition, given their training in that area) and a professional one (committed partnerships).

However, more than half of the field participants said the intervention had been imposed upon them. Because of this, some of them, especially the teachers, were quite reluctant to take part in it, particularly since they had not necessarily identified overweight and obesity as a priority issue within their institutions. This affected their participation and their degree of involvement in the action, which in turn influenced its implementation.

*F15: ‘What immediately put off teachers is that it was a ‘ready-made’ action which they were just supposed to go along with. “Here’s the plan. Now do this and that, like this and like that.” They didn’t like that at all. They would have liked to work on the planned action beforehand, be involved and come up with suggestions, take a little initiative. But in this case, it was not possible. The problem of obesity put them off a little to start with, in addition to the action being parachuted onto them. I think it’s the latter which really bothered them.’*

*S1: ‘It all also depends on how the teams felt, since, in one way, the project was ‘strongly suggested by Headquarters’ to them. So, for some, there was a feeling of being obliged to participate. This made it a negative experience, and it was perceived as being an additional burden regarding which they had not been consulted to start with.’*

That this action was imposed without stakeholder involvement from the beginning of the protocol’s creation, as would have occurred in a true partnership, was widely deplored. According to the field participants, such involvement could have facilitated and enhanced their acceptance of the intervention’s values and process as well as its implementation.

These factors led stakeholders to take various personal positions that might have had an impact on the intervention and its implementation. When participants had either a professional or personal interest in the subject of nutrition, their dismay over not having been more fully involved in the management and coordination of the action did not, however, have an impact on their further involvement. They found other interests and motivations that were external to the action itself. Their standpoint could be professional (work assignment) or due to a personal interest in the topic of nutrition.

*I2: ‘I felt really subordinate, and I had no decision-making role in the running of this project.*

*JT: Did that affect your motivation to participate in the intervention?*

*I2: No, because, in fact … No, personally I liked this project, it was not a barrier.*

*JT: Were there some elements that motivated you to participate?*

*I2: Nothing but the subject matter, which I find very interesting and important.’*

#### Management support

The PRALIMAP implementation was part of the participating schools’ planning over a two-year period and, as such, the establishments’ functioning had to be completely reorganized during the commitment period.

*F8: ‘We must be able to adapt a little bit to students’ planning, their timetable, and find times when they are available so that we can work and get together. It also depends on the manager and the importance he gives to the project. It depends on the person.’*

The school’s head teacher was indeed recognized as a linchpin of the intervention by all stakeholders, with the potential to facilitate or impede the success of the intervention.

*F9: ‘And the fact that—maybe I shouldn’t say this, but this is important—at the steering level, at the management level, we persisted, we did what had to be done for it to work, we really played our steering role. This matters, because if you set the operation aside saying, “It’s interesting, but I have other things to do,” then it’s over.’*

From the discourses of sponsors, intermediary participants, and field participants, it appeared the head teachers’ involvement was a condition for good implementation. The head teachers had to establish and maintain a strong dynamic in the school to sustain the field participants’ motivation to be involved and develop actions. This was very important because PRALIMAP was a long-term program, and a relationship between duration and motivation was observed, especially by field participants.

*JT: ‘The motivation, over time, was it fairly constant?’*

*F11: ‘It has dropped a little, necessarily, because after a year or two it’s okay, but then it starts to…’*

### Transferability-promoting factors and barriers

#### Support to field participants

In the PRALIMAP program, many actors from different spheres (public health, education/research, field, intermediate players) were committed to acting together on the same issue. Because these actors had different cultures, methodologies, and practices, this commitment could, especially for field participants, require major changes in their daily practice. Support provided to field participants through the intermediaries was described as a facilitating factor in the program’s implementation in their school. Indeed, field participants greatly appreciated the mediation between themselves and the sponsors provided by a competent intermediary with prior knowledge of the functioning of schools. This support resulted in more effective collaboration between field participants and sponsors by ensuring that each party’s constraints and imperatives were taken into account. This accompaniment was provided throughout the program’s implementation and helped to reinforce actions taken in the schools and to manage problems in the field.

*F6: ‘The help and the supervision of the PRALIMAP team definitely facilitated things. The protocols also helped.’*

*F11: ‘So I went back to a project manager who came in to provide support. It was really appreciated. Because at least we acquired a method, etc.’*

*F9: ‘These projects related to overall health are really worthwhile, and the support of professionals in this field is very useful to us, because if we’re trying, in this establishment, to take successful action in terms of prevention, information, awareness-raising and education, in many fields, then it’s clear that when we have the support of professionals and of people with expertise, it’s very helpful to us in carrying out our activities and in ensuring that what we are setting up is relevant.’*

*I1: ‘But what was beneficial for the schools, too, was being able to identify a resource person who was outside of the school and who, as such, was able to defuse certain situations that came up in their routine operations and then let the dust settle and move on to other things.’*

This assistance helped increase the participants’ motivation and reinforced their involvement in the program.

#### Team instability

One barrier mentioned was team instability. Indeed, the participant turnover rate, very high in schools, was cited as a major obstacle to implementing the intervention. It was a make-or-break factor, in that those arriving after the project had already started had other priorities related to carrying out their new positions and did not feel involved. The teamwork dynamic broke down, and it became important to establish a relationship of trust with the newcomers in order to start a new process of working together, which in itself hindered the intervention’s implementation.

*I2: ‘Another obstacle is the turnover. You work two years with people. I tell you, the real problem in the projects is people. You have to build a trusting relationship, then the people are replaced or move away, and you have to start again from the beginning. That’s really the difficulty; for me it’s really the second biggest obstacle.’*

## Discussion

This article presents one case study to describe and analyze stakeholders’ perceptions of transferability criteria. Stakeholders’ views on influential processes, from criteria to action mechanisms and outcomes, were collected from their statements in qualitative interviews. Our results showed that many elements with a potential influence on intervention outcomes were addressed by all the stakeholders, although unequally. Three elements were discussed by interviewees more extensively, highlighting the implementation and intervention outcomes: intervention modalities, stakeholder participation, and barriers to transfer. This case study thus outlines three important aspects to consider when implementing and transferring a health promotion intervention.

### The multidisciplinary approach as a major criterion

Our exploration of the hypotheses showed that a multidisciplinary approach was essential to the success of an intervention’s process, both for participants (interdisciplinary teamwork) and for management (supporting this approach). As documented by Kam et al. [32], the manager’s support and involvement in implementing the action is very important, particularly in school-based health promotion programs. Respondents perceived an important principle to be that health promotion is an interdisciplinary team activity, and that no single profession has a monopoly on health promotion knowledge or is equipped to perform all the necessary tasks [33]. In fact, teamwork and team development are prerequisites for effective health promotion outcomes [33]. This multidisciplinary approach must be incorporated into the implementation process, and specific procedures are needed to support participants in implementing this approach.

### Participatory process

Another important element is the notion of participatory process. This includes stakeholder autonomy, i.e., involving field participants in setting goals and giving them the freedom to adapt the intervention when necessary. A top-down program whose objectives are not considered a priority by the stakeholders may lead to low involvement, which is ultimately detrimental to the intervention’s success.

The case study indeed showed a conflict between stakeholders’ perceptions of the action and their sociocultural and professional environments. When the intervention corresponded with the stakeholders’ personal values (considering obesity a health problem; perceiving health education as part of school education), having little or no involvement in developing the intervention protocol had minimal impact on stakeholder involvement. On the other hand, when stakeholders’ professional, cultural, or social expectations were not met by the proposed intervention, their involvement was likely to be weaker. Just as studying the population’s perceptions and social environment is essential when implementing a health promotion program [7, 34], it is equally crucial to consider the social and professional environments of stakeholders likely to be involved in the program. While external factors such as personal backgrounds cannot be completely addressed, it is possible to work on stakeholders’ perceptions of the health problem in question (by taking their views into account when identifying priorities) or of how the program is implemented. Working with them on a real shared diagnosis is more likely to lead to their subsequent buy-in, whereas a ‘turnkey’ program without flexibility could be considered unacceptable and potentially not suited to local needs and constraints. This leads to two main points. First, from the standpoint of intervention process and ethics, a health promotion program generally cannot be implemented effectively without involving field participants in the planning process. Second, from an efficiency standpoint, the concept of transferability is based on the fact that the result is a product of the interaction between intervention and context [11], such that any intervention generally must be adapted to its particular context. A top-down program is likely destined to fail due to professional and/or population non-adherence. As underlined by Potvin et al., it would be contrary to some of the core values of health promotion to implement a program without considering the context, the stakeholders involved, and the population. Indeed, health promotion values and principles consider empowerment and community participation to be as important as the health results [35].

### The ASTAIRE tool and support for transferability

ASTAIRE is a tool that was developed by experts to analyze the transferability of health promotion interventions. It is useful for comparing settings, guiding the choice of the primary intervention most suited to the replica setting, and, if necessary, supporting the intervention’s adaptation to that specific setting. It can complement pre-existing frameworks, guidelines, and tools in the fields of health promotion or health care, and generally focuses on applicability and implementation criteria [36–39]. In this study, ASTAIRE served as a reference framework for discussing transferability with health promotion program stakeholders. The present case study represents one testing ground for the tool; it would need further testing, on case studies presenting other populations, other settings, and other health promotion programs.

Another major issue when transferring an intervention is support for knowledge transfer. Indeed, in evidence-based health promotion processes, one cannot move from research (or innovation) to real-life practice without supporting stakeholders in the transfer. This transfer is not one-way; it has to involve mutual learning between stakeholders and researchers (scientists learning from the experience of field workers) [40, 41]. To facilitate this process, new forms of knowledge transfer, such as knowledge brokering, have been tried. This mediation is intended to create links between researchers and stakeholders to facilitate their interaction, help them understand the objectives and the specific ways in which the different professional cultures function, enable them to influence each other’s work, create new partnerships, and promote the use of research evidence [42, 43].

### Limitations

The objective of this case study was to analyze the perceptions of stakeholders; those of the population were not considered. It therefore offers only part of the picture and would need to be supplemented by further research, including case studies on different health promotion interventions, with a focus on the perspectives of both stakeholders and the population. Indeed, this case study was the first analytical case to adopt a realist approach [44]. Other case studies are currently under way, based on other health promotion interventions, with the objective of further examining and modifying the hypotheses regarding population-related transferability criteria. This study is part of a larger project that addresses evidence-based health promotion by maximizing the transferability of results with respect to public health decisions and interventions. Stakeholders’ perceptions are especially important to understand what criteria they identify as influencing an action’s results. Indeed, the implementation process and program transferability depend on stakeholders’ perceptions of particular criteria. This kind of diagnosis is therefore important in providing feedback so that tools, procedures, and training can be appropriately adjusted.

Another limitation was in the choice of intervention for this case study. The PRALIMAP intervention was a ‘turnkey’ program imposed on stakeholders in a research context; the program’s evaluation conditions required scrupulous application of procedures with no possibility of adapting them and without regard to environmental and population characteristics. This could have influenced the respondents’ statements regarding aspects of implementation, to the detriment of contextual criteria.

## Conclusion

The transferability of health promotion interventions is a particularly important consideration for researchers and stakeholders, but paradoxically has been relatively unexplored [11, 22].

Our work contributes to a better understanding not only of transferability factors, but also of stakeholders’ perceptions about them, which is just as important, because those perceptions themselves are a factor in mobilization, implementation, and transferability. On a practical level, our results encourage researchers and developers of interventions to be especially vigilant regarding several factors, especially multidisciplinary teamwork, the involvement of stakeholders in the process, and support for transfer. Pursuing this research through other case studies will be helpful for refining these findings, proposing possible adaptations of ASTAIRE, and developing broader processes for implementing interventions in specific contexts.
